# Autonomic Stress Response and Perceived Effort Jointly Inform on Dual Tasking in Aging

**DOI:** 10.3390/brainsci9110290

**Published:** 2019-10-24

**Authors:** Giancarlo Condello, Roberta Forte, Pablo Monteagudo, Barbara Ghinassi, Angela Di Baldassarre, Laura Capranica, Caterina Pesce

**Affiliations:** 1 Graduate Institute of Sports Training, Institute of Sports Sciences, University of Taipei, Administrative Building, 101 Zhongcheng Rd. Section 2, Shilin District, 111 Taipei, Taiwan; giancarlo.condello@gmail.com; 2 Department of Movement, Human and Health Sciences, University of Rome Foro Italico, Piazza Lauro De Bosis 6, 00135 Roma, Italy; roberta.forte@uniroma4.it (R.F.); laura.capranica@uniroma4.it (L.C.); 3 Department of Physical Education and Sport, University of Valencia, Av. de Blasco Ibáñez, 13, 46010 València, Spain; Pablo.Monteagudo@uv.es; 4 Department of Medicine and Aging Sciences, “G. d’Annunzio” University of Chieti-Pescara, Via dei Vestini 31, 66100 Chieti, Italy; b.ghinassi@unich.it (B.G.); a.dibaldassarre@unich.it (A.D.B.)

**Keywords:** alpha-amylase, RPE, dual-task, working memory, physical activity

## Abstract

The study investigated, through neuroendocrinological, subjective and behavioral assessments, how aging individuals cope with locomotor-cognitive dual-tasking and whether physical activity habits influence the acute response to locomotor-cognitive performance. Seventy-nine healthy participants aged 55–85 years were assessed on locomotor (gait speed, stride length) and cognitive (working memory) performances under single- and dual-task (ST, DT) conditions, and habitual physical activity (daily steps). Rating of perceived exertion (RPE) was assessed immediately after performance. Salivary α-amylase (sAA) was measured prior, immediately and 5 min after performance. Gait and working memory variables, the area under the curve of sAA (AUC) and DT–ST differences (DT effects) were computed. AUC was higher when the ST or DT performance involved a locomotor component and showed a pre-to-post increment after DT only, whereas RPE was higher when performance involved a cognitive component. Daily steps neither predicted sAA, nor RPE. Associations between DT effects on sAA, RPE and performance emerged in high-active participants only. In aging individuals, DT walking elicits an autonomic stress response presumably led by the challenge to share resources relying upon common neural substrates. This autonomic response seems tuned to gait performance and subjective evaluation of effort in those more accustomed to walking.

## 1. Introduction

With aging, gait seems no longer automated and walking becomes cognitively demanding [1]. This is particularly evident when older individuals perform a concomitant cognitive task while walking, as indicated by the larger decrement in gait performance from single-task (ST) to dual-task (DT) conditions compared to younger adults [2]. Furthermore, the performance decrement from ST to DT is proportional to the extent to which DT walking is perceived as stressful [3]. In turn, acute stress seems detrimental to the ability to shield performance of a primary task when a secondary one must be performed concurrently. This is attributed to reduced efficiency of the cognitive control mechanisms responsible for resource allocation needed for successful DT performance [4]. Indeed, these mechanisms rely on frontal-dependent cognition—the executive control network—some of which functions, as working memory is impaired by stress [5].

There is a range of subjective and objective approaches and tools to assess stress-related responses, resource allocation and effort in coping with physically and/or mentally challenging tasks. Subjective indexes of the perceptual and cognitive responsiveness to task performance, such as the rating of perceived exertion (RPE) [6], may complement physiological measures for a comprehensive understanding of the responses to physical-cognitive DT performance. 

Among objective and non-invasive measurement, saliva sampling provides biomarkers relying on the two main systems that jointly contribute to psycho-physiologic stress responses: cortisol and α-amylase [7]. Cortisol is secreted from the adrenal cortex via the hypothalamic-pituitary-adrenal axis, whereas α-amylase is secreted by the parotid gland in response to adrenergic activity [8]. 

Salivary α-amylase (sAA) seems a more sensitive and generalizable stress response marker than cortisol, as it reacts more pronouncedly and rapidly to different types of stressors and returns to baseline levels immediately after the end of the stress bout without the carry-over effect observed for cortisol [9]. Thus, sAA has been increasingly used as a valid biomarker of autonomic stress response within the sympathetic adrenomedullary system [10]. Its stress responsiveness has been proven in different age groups throughout the lifespan including older adulthood [11,12]. Aging tends to heighten the sympathetic tone, as revealed by a higher sAA global output than in younger adults [13], without attenuating the autonomic response to stress, as revealed by similar stress-related increases of sAA in younger and older adults [14]. 

Studies of sAA response to physical stressors have mainly investigated the impact of physical exercise bouts of long duration (>20 min) and moderate-to-high intensity (>50% maximal oxygen consumption), showing that they acutely elevate sAA [15]. Instead, the few studies on physically low demanding exercise (e.g., light self-paced walk) did not show any significant increase in sAA concentration [16]. 

On the other hand, sAA response to physical exercise tasks has been rarely studied in relation to task difficulty and task-related cognitive challenges under ST and DT conditions [17,18]. This is surprising, since there are neural commonalities between cognitive control in dual-tasking and biological acute stress responses, which become particularly relevant in aging. The cognitive control processes responsible for DT performance and task prioritization have been located in the prefrontal cortex [19]. Prefrontal neurons, and particularly those engaged in working memory, are influenced by the activation of the two main stress axes responsible for an increased release of catecholamine and cortisol [20,21]. This is relevant to the present study, because working memory seems more relevant to gait efficiency and resource allocation during DT walking in aging than the other core components of prefrontal-dependent cognition [22,23]. 

Moreover, there is evidence that habitual physical activity and/or fitness may positively influence the ability to cope with dual-tasking [24]. Habitually highly active and fit individuals also show, in the majority of studies, reduced physiologic stress responses [25]. This is consistent with the hypothesis that regular physical activity may lead to physiological adaptations that buffer the effects of stressors in physical and non-physical domains (the cross-stressor adaptation hypothesis) [26]. However, the most used bio-physiological indicators of stress response were cortisol concentration or cardiovascular parameters of autonomic activity. The evidence on sAA is inconsistent and limited to young and middle-aged individuals’ responses to social-evaluative stressors [27,28]. A recent study failed to find moderation of the acute sAA response to stressors by habitual physical activity levels [29].

Thus, in consideration of the above lack or inconsistency of evidence, the aim of the present study was twofold. The first aim was to investigate how aging individuals cope with locomotor-cognitive dual-tasking by analyzing complementary information on their autonomic response to DT performance and cognitive effort [6,11,17]. Given the relevance of working memory for gait performance of older individuals [22], it was hypothesized that a working memory task would compete for shared resources with the walking task. We expected a higher objective response and subjective effort perception to the locomotor-cognitive DT as compared to the locomotor or cognitive task performed in isolation. Most aging research on locomotor-cognitive dual-tasking included participants aged above 60 years with comparison groups mostly aged below 40, whereas middle age is relatively understudied [2]. To extend the evidence base to the transition to older adulthood, we included both late middle-aged and older adults. 

The second aim was to investigate whether habitual physical activity, measured as daily steps [30], influences the autonomic response to locomotor-cognitive dual-tasking and its tuning to the subjective perception of effort. We tested whether the absence of moderation of sAA responsiveness by physical activity level found in young adults [29] generalizes to late-middle-aged and older adults. Alternatively, according to the cross-stressor adaptation hypothesis, being active may reduce the autonomic response to locomotor-cognitive DT performance (i.e., lower change in sAA concentration and faster return to baseline). Furthermore, chronic physical activity increases the individual’s ability to tune perception of effort with task demands [6]. Thus, we hypothesized that the perception of effort and autonomic response of higher active individuals would be better aligned with their behavioral task performance. 

## 2. Materials and Methods

This study was approved by the Ethics Committee Azienda Policlinico Umberto I (Rome, Italy, reference number: Prot. 451/13). All participants, recruited through flyers and presentation of the study in different senior centers and trade unions, gave written informed consent in accordance with the Declaration of Helsinki. 

### 2.1. Participants

The sample consisted of 79 individuals (F = 36, M = 43) ranging in age from 55 to 85 years, divided into late-middle-aged (55–64 years: *n* = 35) and older adult (≥65 years: *n* = 44). They answered the AAHPERD exercise/medical history questionnaire [31] to ascertain their physical activity and health history, including medication use, dietary habits, tobacco smoking/alcohol consumption, the presence of cardiovascular, pulmonary, muscular-skeletal, sensory and metabolic conditions/diseases (Table 1). Inclusion criteria were BMI <35 kg·m^−2^, absence of eating disorders, alcohol/substance abuse, pathological conditions that could influence study outcomes—such as thyroid dysfunction and other endocrine/metabolic conditions—and absence of known history of cardiac illness and cerebrovascular disease. Prior to the evaluation, participants were asked about their educational background and screened for cognitive executive function by means of the trail making test. This paper-and-pencil test has been widely used for its sensitivity to detect any kind of brain damage [32]. It comprises a part A, assessing visual-motor tracking and sustained attention, and a part B, additionally requiring task alternation ability. The difference in time between parts A and B is considered a measure of cognitive flexibility relatively independent of manual dexterity. In general, participants’ performance was above average but within one standard deviation (SD) of the mean of national reference data for similar age groups [32], except for the oldest higher active participants, who were higher than the one SD threshold (Table 1).

### 2.2. Procedures

Participants were tested individually for anthropometric measurements, habitual physical activity level, locomotor and cognitive performances under single- and dual-task conditions. Standing height to the nearest 0.1 cm and body mass to the nearest 0.1 kg were measured using a portable stadiometer (Seca 220, GmbH & Co., Hamburg, Germany) and a balance scale (Seca 761, GmbH & Co., Hamburg, Germany), respectively. Body mass index (BMI, kg·m^−2^) was computed.

### 2.3. Physical Activity Level

The habitual physical activity level of participants was measured under free-living conditions using the SenseWear Pro3 Armband (BodyMedia, Pittsburgh, PA, USA). The use of SenseWear Pro Armband has been already validated in older adults [33]. The armband is a monitor that integrates the information gathered by the two-axis accelerometers and sensors (e.g., skin and near body temperature, heat flux and galvanic skin response) with sex, age, height, weight, smoking status and handedness of the user. It provides proprietary algorithms to give quantitative information (e.g., number of daily steps, locomotor activity intensity and energy expenditure; [34]) about an individual’s habitual physical activity involving any form of locomotion as activities at workplace, sports, conditioning and household. The descriptive characteristics of the participants were entered into the software program (SenseWear Professional 8; BodyMedia, Pittsburgh, PA, USA) before the monitoring was initiated. The participants wore the armband on the right arm over the triceps muscle at the midpoint between the acromion and olecranon processes. According to reliability criteria reported in the literature, participants wore the armband for seven entire and consecutive days, 24 h a day except during water-based activities [35], with a wear time of at least 540 min/day on weekdays and 480 min/day on weekend days [34]. From the default information given by the software, the mean value of steps of 7 days was used for the statistical analysis. According to international age-related norms [30], participants were categorized as lower active (low or somewhat active, ≤9999 steps/day) and higher active (active or highly active, ≥10,000 steps/day). Since the lower active group took on average almost 8000 steps/day (Table 1), with only one participant with 4,903 daily steps felling below the 5000 steps/day threshold of sedentary classification, most of the participants were quite active and fit. 

### 2.4. Motor-Cognitive Performances

Participants were tested on three task conditions: locomotor ST, cognitive ST and locomotor-cognitive DT. The locomotor performance under ST condition consisted of a habitual walking speed (HWS) trial around a rectangular path of 10 × 2 m for 2.5 min. Gait analysis was performed using a 10 m photocell system (Optojump Next, Microgate, Bolzano, Italy) consisting of 10 transmitting and 10 receiving optical bars (each containing 96 LEDs) placed parallel to each other at 2 m distance. The LEDs on the transmitting bar communicate continuously with those on the receiving bar. The system detects any interruptions in communication between the bars and calculates their duration, to measure parameters connected to gait performance. To consider a steady-state gate performance, the data from the first and last 1 m bars were excluded from the analysis, thus avoiding the influence of acceleration and deceleration phases. Quantitative gait parameters were calculated by the system (Optojump Next, Microgate, Bolzano, Italy Software version 1.9.7.0) to obtain mean values of speed [m·s^−1^] and stride length [m] (the mean value of stride length normalized for height) and respective coefficients of variation (CV: ratio of standard deviation (SD) to the mean per 100). The choice of the parameters is based on literature indicating gait speed and stride length as good parameters for assessment in older individuals [36].

The cognitive performance under the ST condition consisted of the random number generation (RNG) task. The RNG is a complex working memory task that requires to generate random number sequences. Keeping track of recent responses and comparing them to a concept of randomness, which is a central aspect of RNG, requires the updating ability to monitor response distribution while suppressing stereotyped responses [37]. This task is believed to reflect executive processing functions that depend on the integrity of the prefrontal cortex [38]. It is a multidimensional test, which is feasible with older adults [39]. The test required participants, who were seated in a quiet room, to verbally generate a sequence of 100 numbers, chosen randomly between 1 and 9, at a frequency of 40 bpm paced by a metronome. A practice trial always preceded the test. The generated numbers were manually and electronically recorded to elaborate the randomness of the sequence (https://www.lancaster.ac.uk/staff/towse/rgcpage.html). Three indices reflecting working memory updating were obtained: the redundancy (Red), the coupon score (Coupon) and the mean repetition gap (MeanRG). The Red index reflects the unbalance of response alternative frequencies in a sequence that derives from a more frequent usage of given numbers than expected based on the theoretical frequency of each digit in random responses. The Coupon is the measure of the mean number of responses given before all the alternative responses are used. The MeanRG is the average quantity of digits between successive occurrences of the same number calculated for all digits throughout the whole sequence. Data were z-standardized, Red and Coupon values reversed to calculate a summary index of Red, Coupon and MeanRG, with higher values corresponding to better memory updating performance.

The locomotor-cognitive DT condition consisted of the combination of the locomotor walking task whilst performing the RNG. For this condition, both the gait parameters and the RNG indexes were considered. To obtain indexes of DT interference from gait performance data, we calculated relative dual-task effects (DTE) as follows [40,41]:

$$\mathrm{DTE}=\left[\frac{\left(\mathrm{dual}\;\mathrm{task}-\mathrm{single}\;\mathrm{task}\right)}{\mathrm{single}\;\mathrm{task}}\right]\times 100\%.$$

In this way, negative DTE values indicate deteriorated performance in DT (i.e., dual-task cost), whereas positive values represent an improvement in DT with respect to ST (i.e., dual-task benefit). To obtain a performance index of DT interference from cognitive performance data, the relative DTE formula above could not be used because the summary index of working memory updating was z-standardized. Thus, we calculated the difference score as absolute DT–ST performance.

Each task condition lasted 150 s with a 15 min rest in between. A familiarization session was organized one week prior to the experimental one. Quality control of the task conditions was ensured by means of video recordings. The three task conditions were randomly administered among participants to avoid practice and accumulated fatigue effects. 

### 2.5. Rating of Perceived Exertion

At the end of each task condition, participants rated their perceived exertion (RPE) by using the Borg CR10-scale [42]. This is a category–ratio scale anchored at the number 10, which represents extreme effort. To obtain indexes of DT effects on perceived effort, DT–ST differences in RPE were computed to estimate the change in perceived effort elicited both by adding the cognitive task to walking (DT–ST walking) and by adding the walking task to random number generation (DT–ST random number generation).

### 2.6. Saliva Sampling

Unstimulated saliva samplings (>0.05 μL) were obtained by means of cotton swabs and saliva collecting tubes (Salivette, Sarstedt, Germany) before (Pre), at the end (Post) and at 5 min (Post5) of the recovery phase of each task condition for the analysis of sAA. The participants were instructed to place the cotton swab into their mouths for 2 min and to chew 20 times, under the supervision of an investigator. The absence of blood contamination was checked with a salivary blood contamination kit (Salimetrics LLC, State College, PA, USA). The saliva collecting tubes were centrifuged at 3000 rev·min^−1^ for 15 min at 4 °C, saliva samples were then stored at −80 °C until they were assayed. To avoid any variations between tests, all samples were tested in the same series and in duplicate by means of a kinetic reaction assay kit (Salimetrics, LLC, USA). The intra-assay coefficient of variation and inter-assay reproducibility of 5.47% ± 1.49% and 4.7% ± 0.15% were accepted, respectively. A standard plate reader (PowerWave XS, Bio-Tech Instruments, USA) was used for salivary determination by 405 nm filters. For each test condition, the area under the curve with respect to ground (AUC_G_) was calculated [43] and used to analyze task condition effects on sAA global output. To analyze the time course of the autonomic response, sAA concentration data at each measurement point under the different test conditions were used. To obtain indexes of DT effects on the autonomic activity, DT–ST differences in AUC_G_ were computed to estimate the change in sAA elicited both by adding the cognitive task to walking (DT–ST walking) and by adding the walking task to random number generation (DT–ST random number generation).

### 2.7. Statistical Analysis

Data were analyzed using the Statistical Package for the Social Science, version 24.0 (SPSS Inc., Chicago, Illinois). The level of statistical significance was set at *p* < 0.05 for all computations. A predefined outlier management policy was applied to avoid the risk of distortion of parameter estimates. The Mahalanobis distance was used for detecting and excluding outliers from the multivariate data setting as long as they were not systematically associated with specific background characteristics. All sAA data (sAA concentration at each measurement point and AUC_G_) were log-transformed due to non-normality (tested with Shapiro–Wilk).

To investigate differences in sAA global output among the three task conditions (e.g., locomotor ST, cognitive ST and locomotor-cognitive DT), a mixed model ANOVA was applied to AUC_G_ data with task condition as within-participants factor and age (late-middle-aged vs. old) as between-participants factors. The same model with the addition of the factor timing was run on sAA concentration data at each measurement point to investigate the time course pre, post and 5 min after performance of locomotor ST, cognitive ST and locomotor-cognitive DT. Effects sizes were calculated as partial eta squared (*η*_𝑝_^2^) for ANOVA results. Post-hoc analysis was performed with planned *t*-tests. 

To investigate differences in perceived effort among the three ST and DT conditions, RPE rank data were submitted to Friedman non-parametric, repeated-measures ANOVA with task condition (locomotor ST, cognitive ST, locomotor-cognitive DT) as factor. Wilcoxon test for paired samples was run for post-hoc pairwise comparisons between task conditions. To evaluate the eventual moderation by age class, the same analysis was performed separately for late-middle-aged and old adults. Bonferroni correction was applied to all parametric and non-parametric post-hoc tests according to the number of multiple comparisons.

To explore the influence of physical activity habits on the objective autonomic response and subjective effort perception elicited by ST and DT challenges, regression analyses were performed. AUC_G_ and RPE data were regressed on daily steps separately for locomotor ST, cognitive ST and locomotor-cognitive DT condition. Linear and ordinal regressions were run for AUC_G_ and RPE data, respectively. Since daily steps were negatively associated with age (r = 0.310, *p* = 0.005), age was entered in the regressions as a covariate. 

To explore the strength of the association between autonomic response to DT, effort perception and behavioral DT performance in lower active and higher active individuals, correlational analysis was performed among DTE variables: Pearson’s r for AUC_G_, gait parameters (mean and CV speed and stride length) and working memory (summary index) data and Spearman’s rho for RPE data. Correlations were computed both on the entire sample and separately on the two subgroups of lower active and higher active participants.

## 3. Results

The Mahalanobis distance detected three outliers in the sample when the variables sAA and RPE under the three task conditions were considered. These outliers were not homogeneous in any relevant background variable (age, gender, education level, activity level, health conditions/diseases and executive function). Thus, they were removed from the sample (Table 1).

### 3.1. sAA Responsiveness to Task Conditions 

**Figure 1:**
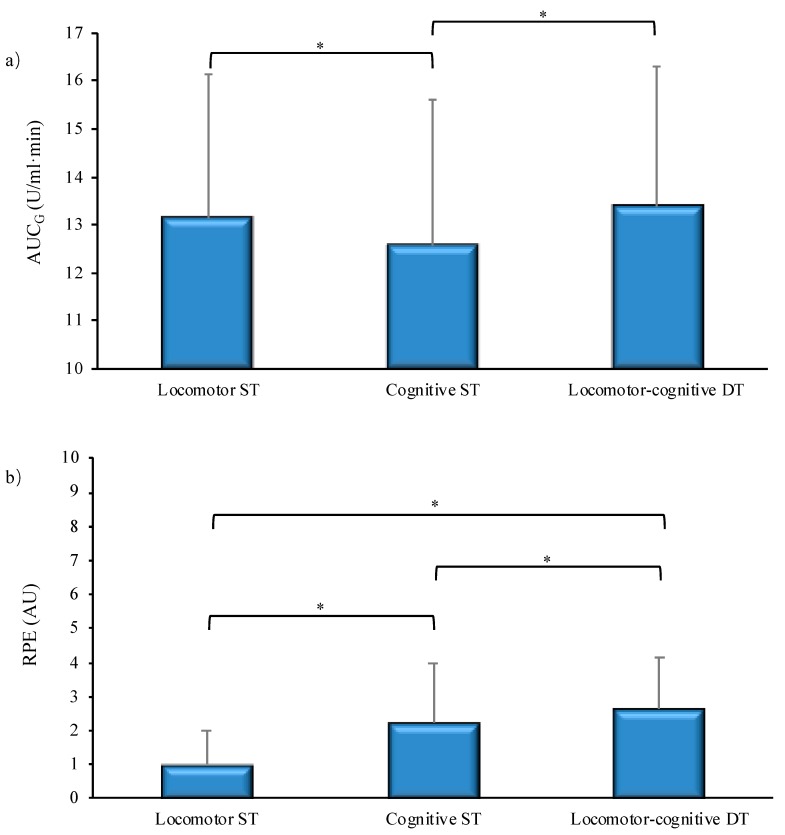
Differences in salivary α-amylase (area under the curve with respect to ground, AUC_G_; panel **a**) and Rating of Perceived Exertion (RPE; panel **b**) among the three task conditions. Locomotor ST = walking task at habitual walking speed (HWS). Cognitive ST = Random Number Generation (RNG) task. Locomotor-cognitive DT = walking at HWS while performing the RNG task. * *p* < 0.05.

From the ANOVA performed on the AUC_G_ of sAA concentration, a significant main effect (with Huynh–Feldt correction of df for ε = 0.95: F (1.9,140.7) = 14.61, *p* < 0.001; *η*_𝑝_^2^ = 0.16) emerged for task condition. Post-hoc analysis (adjusted *p* = 0.016 for three pairwise comparisons) revealed significant differences for cognitive ST (12.6 ± 3.1 U/mL·min) compared to locomotor ST (13.1 ± 3 U/mL·min) and locomotor-cognitive DT (13.4 ± 2.9 U/mL·min) conditions (Figure 1a). No significant interactions of task condition with age emerged.

### 3.2. RPE Responsiveness to Task Conditions 

The non-parametric ANOVA (Friedman’s test) performed on RPE scores revealed a significant effect for task condition both for the entire sample (*p* < 0.001) and separately for late-middle-aged and old adults (*p* < 0.001). Post-hoc analysis (Wilcoxon test; adjusted *p* = 0.016 for three pairwise comparisons) revealed significant differences in RPE among all three task conditions (Figure 1b): locomotor ST (1.0 ± 1.0 AU) vs. cognitive ST (2.3 ± 1.7 AU; *p* < 0.001); locomotor ST vs. locomotor-cognitive DT (2.7 ± 1.5 AU; *p* < 0.001); and cognitive ST vs. locomotor-cognitive DT (*p* = 0.006).

### 3.3. Time Course of sAA Response to Task Conditions

**Figure 2:**
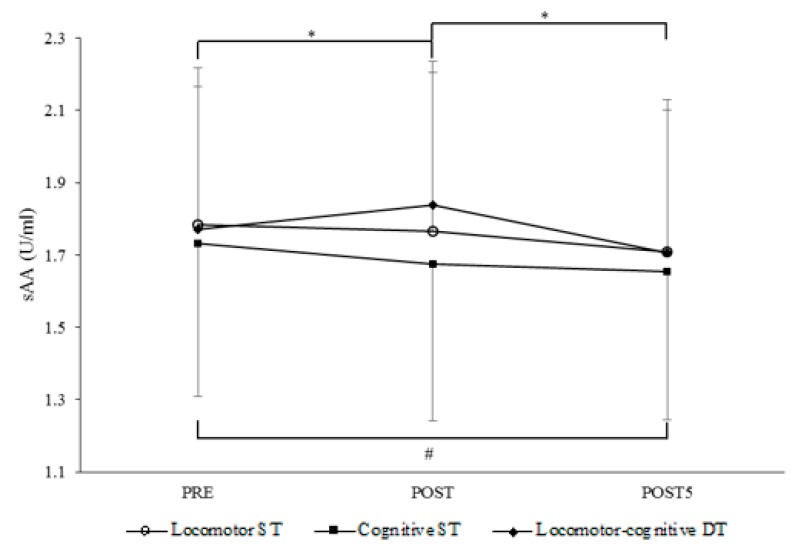
Differences in salivary α-amylase (sAA, log-transformed) sampled before (Pre), immediately and 5 min after (Post, Post5) task performance separately for the three task conditions. Locomotor ST = walking task at habitual walking speed (HWS). Cognitive ST = random number generation (RNG) task. Locomotor-cognitive DT = walking at HWS while performing the RNG task. * and # = significant (*p* < 0.01) differences for locomotor-cognitive DT and for cognitive ST, respectively.

The analysis of sAA concentration at each measurement point showed a main effect for timing of saliva sampling (without Huynh–Feldt correction of df for ε = 1.00; F (2,146) = 17.72, *p* < 0.001; *η*_𝑝_^2^ = 19; linear: *p* < 0.001; *η*_𝑝_^2^ = 0.25; quadratic: *p* = 0.003; *η*_𝑝_^2^ = 0.12) and confirmed the main effect for task condition (with Huynh–Feldt correction of df for ε = 0.94: F(1.9,139.6) = 9.87, *p* < 0.001; *η*_𝑝_^2^ = 0.12) found in the analysis of AUC_G_ data. Moreover, a significant interaction between timing of saliva sampling and task condition was found (with Huynh–Feldt correction of df for ε = 0.94; F(3.9,289.9) = 3.62, *p* = 0.007; *η*_𝑝_^2^ = 0.05). Post-hoc analysis (adjusted *p* = 0.005 for nine pairwise comparisons) revealed significant differences for cognitive ST condition between Pre (1.73 ± 0.43 U/mL) and Post5 sampling (1.65 ± 0.41 U/mL; *p* = 0.003), and for locomotor-cognitive DT condition between Pre (1.77 ± 0.45 U/mL) and Post (1.84 ± 0.40 U/mL; *p* = 0.003), and between Post and Post5 sampling (1.71 ± 0.40 U/mL; *p* < 0.001). This pattern of results is depicted in Figure 2. No significant interactions of timing and task condition with age emerged.

### 3.4. Influence of Physical Activity Levels on sAA and RPE Responses to Task Conditions 

Regression analyses did not yield any significant prediction by daily steps (R^2^s < 0.03) of AUC_G_ or RPE data after controlling for age (AUC_G_: *p* = 0.642, 0.775 and 0.859 for locomotor ST, cognitive ST and locomotor-cognitive DT, respectively; RPE: *p* = 0.615, 0.821 and 0.970, respectively).

### 3.5. Interrelations between Dual-Task Effects on Performance and sAA/RPE Responses 

Correlations were computed on the entire sample and separately on the two subgroups of lower and higher active participants. They revealed significant associations in the higher active group only. Regarding gait performance, only CV speed, but not mean speed or mean/CV stride, was found sensitive to this kind of analysis. DTE on CV speed was significantly associated with DTE on both AUC_G_ and RPE responses, which were not associated with each other (Table 2, top). As regards cognitive performance, DTE on working memory was associated with DTE on RPE but not on AUC_G_; these latter were associated with each other (Table 2, bottom).

## 4. Discussion

This study was developed at the intersection of neuroendocrinological stress research, functional mobility research and acute exercise and cognition research. The main aim was to further our understanding of how lower and higher active aging individuals cope with locomotor-cognitive dual-tasking by means of a joint analysis of autonomic and subjective responses to behavioral DT performance. Neural commonalities exist between cognitive control in dual-tasking and autonomic acute stress responses [20,21]. In light of this, we suggested that the autonomic responsiveness to DT and effort perception, along with reciprocal DT effects of concomitant locomotor and cognitive task performances, would provide complementary information on how aging individuals cope with and react to the challenges of dual-tasking [17]. Moreover, we tested whether, at late-middle-age and old age, regular physical activity may act as a stress-buffer that transfers across domains (i.e., cross-stressor adaptation hypothesis) [26]. We hypothesized that if sAA were a sensitive biomarker of this, higher active aging individuals would show an autonomic response to task demands that is lower, faster returning to baseline and more finely tuned with performance efficiency and effort perception.

Taken together, the results indicate consistently higher objective and subjective responses to locomotor-cognitive DT, which was the only task condition showing an increment in sAA concentration immediately after performance, as compared to ST conditions. Neither the sAA response, nor the subjective perception of effort differed depending on whether aging individuals were more or less active. Instead, we found some support to our hypothesis that in higher active individuals, effort perception and autonomic response to complex locomotor-cognitive tasks are more finely tuned with each other and with the performance cost paid under DT conditions. 

Due to the short duration and low intensity of the walking bouts, we did not expect a neurophysiological modulation in response to the metabolic demands of physical exercise as reported in acute exercise and cognition research for more intense and longer exercise bouts [44]. Rather, we expected the autonomic response and perception of effort to be higher in the case of DT walking, due to the stressful demands of performing a locomotor and a cognitive task concomitantly. The stressfulness of DT walking for aging individuals is corroborated by evidence that the disproportional age-related deterioration in gait during DT compared to ST walking [23] is associated with increased fall risk and fear of fall, which in turn generates the conditions for an increased likelihood of falling [45,46]. 

The results confirmed that dual-tasking elicited highest autonomic stress response and perceived effort (Figure 1a,b). However, the size of the subjective and objective responses diverged for single-tasking. It seems that the cognitive task demands made a major contribution to the subjective evaluation of effort (Figure 1b), probably due to the very light and therefore subjectively negligible intensity of a short bout of walking at habitual speed. The autonomic responsiveness, instead, was higher in the locomotor ST or DT conditions with respect to the cognitive-only condition (Figure 1a). This reverse pattern is the result of different trends of sAA concentration across measurement points under the three task conditions (Figure 2). There was an increment in sAA during the locomotor-cognitive DT only, which rapidly returned to baseline 5 min post-task. This is in line with evidence that sAA rapidly increases with the exposure to a stressor and returns to pre-stress levels immediately after its end [47]. In contrast to the DT condition, there was no significant change after the locomotor ST and a decremental trend in sAA concentration immediately after the cognitive ST. Speculatively, the higher sAA response to the locomotor ST with respect to the cognitive ST may be due to the fact that walking at habitual speed, though requiring only very low energy expenditure, is a dynamic activity requiring muscular activation and balance control. Instead, the RNG task was performed sitting quietly, with no additional demands.

Physical exercise is proven to acutely elevate sAA in a dose–response fashion, as suggested by a progressively lower sAA response to running, light jogging and walking [48] that becomes non-significant for low demanding locomotion, as self-paced one-time walking [16]. In this study, we did not manipulate the physical exertion of the locomotor task, which in ST condition fell below a presumable threshold of effort necessary for inducing an autonomic response. Conversely, we manipulated task effortfulness and cognitive control demands in a way that mirrors concomitant walking and thinking aging people face in their everyday life. The main challenge that presumably led to the autonomic response was resource sharing because the locomotor and concomitant working memory task relied upon common resources and neural substrates in the prefrontal cortex [22].

It is worthy to consider the threefold role of prefrontal neurons engaged in working memory that may influence and be influenced by the autonomic response to our locomotor-cognitive DT. Firstly, working memory and the underlying prefrontal cortex have been identified as the locus of the cognitive control processes involved in DT gait control in aging [23]. Particularly, the dorsolateral prefrontal cortex seems involved in processes of task prioritization in DT [19]. Secondly, the RNG has been developed as a task challenging the executive control of working memory [38]. Indeed, the working memory updating function has a central role in RNG by keeping track of recent responses and comparing them to a conception of randomness [37]. Neuroscientific evidence also highlights the particular involvement of the dorsolateral prefrontal cortex in the suppression of counting routines to generate randomness [49]. Thirdly, the prefrontal neurons engaged in working memory seem, in turn, negatively influenced by stress-induced increases in sympathetic nervous system activity. A high release of catecholamine that is induced by the autonomic response to stress, coupled with an increase in cortisol secretion, seems to diminish the firing rates of prefrontal neurons and especially those in the dorsolateral prefrontal cortex [20,21].

The results of our study did not support the hypothesis that practicing regular physical activity buffers and reduces the time course of the autonomic response to acute stressors in physical and cognitive domains (cross-stressor adaptation hypothesis) [26]. This generalizes the notion that sAA stress responses are not influenced by habitual physical activity from younger adulthood [29] to late-middle-aged and older adulthood. In fact, the number of daily steps habitually performed did not explain inter-individual differences in autonomic response to either of the locomotor, cognitive or locomotor-cognitive tasks. 

Conversely, in line with our expectation, we found moderate associations of the autonomic response to the challenge of DT walking with the perceived exertion and overt performance. This evidence emerged in persons who were active or very active but not in those who were low or only somewhat active (Table 2). It seems supportive of the hypothesis that higher active individuals are better able to utilize intrinsic feedback [6] to tune the perception of effort with task demands. 

The analysis of the reciprocal DT effects of the walking task on cognition and of the cognitive task on walking provided some support for a more nuanced interpretation of the pattern of objective and subjective responses to DT challenges. Associated increments in autonomic response and perception of effort were found in higher active individuals when their cognitive performance was challenged by a concomitant walking task (Table 2, bottom). Due to their walking habit, they seem able to tune their autonomic reaction to the estimation of the cost—in terms of effortful resource allocation—of performing a cognitive task while walking. All participants, independently from their physical activity levels, perceived that performing the cognitive task while walking was more effortful than performing it in single fashion (Figure 1b). However, only higher active individuals showed an increase in effort perception proportional to their autonomic response and to the decrement in cognitive performance (Table 2, bottom). 

The subjective effort perception was found associated with both the cognitive and the locomotor cost of dual-tasking. Since it was observed in higher active individuals only (Table 2), it might be suggested that the effort perceived in the physical and cognitive domains may not necessarily converge into a unitary construct that generalizes to the overall aging population. It is an actual issue of debate whether cognitively experienced effort is a unitary construct. Westbrook and Braver [50] discussed the view that the neural systems, which originally emerged to monitor physical effort, may have evolved into cognitive load monitors. They intriguingly argued that the demands underlying physical actions may have no analog in the demands of cognitive processes. In light of the differential results of our lower and higher active subgroups, we speculate that the domain-generality or specificity may also depend on the individual ability to deal with the unique demands of physical actions.

The correlational analyses also inform on which aspect of the locomotor performance, deteriorated by a concomitant cognitive task, is associated with objective and subjective responses in higher active individuals (Table 2, top). The more the resource sharing between gait control and cognitive performance disrupts the stability of gait speed, the more individuals accustomed to walking seem to perceive the DT as effortful and respond physiologically with a higher autonomic stress response. Gait speed, whose variability is especially sensitive to locomotor-cognitive interference [2] is a clinically relevant gait parameter linked to several health outcomes as the maintenance of balance or falls [51]. This finding may have implications for fall prevention in complex DT walking. Being able to perceive a level of effort proportional to one’s difficulty in maintaining stable gait speed might prevent higher active aging individuals from underestimating fall risk and the potentially disruptive effect of complex dual tasking on gait. However, not only correlational studies are needed to corroborate this speculation.

The study has limitations to be addressed. Huge inter-individual variability in sAA data was observed. A certain degree of inter-individual variability is often reported [29,52]. It can be partially ascribed to personal factors potentially interacting with situational characteristics and stressor type. Nevertheless, a source of the high variability in the present data set can be the assumed impact of tactile and salivatory factors on sAA secretion, which is a weakness of Salivettes’ use for the measurement of sAA concentration [11]. This may have weakened the observed effects and led to some nonsignificant correlations of sAA with perceived effort and locomotor-cognitive performance, which are in opposite directions for low and high activity individuals and difficult to interpret. Another limitation regards the overall physical activity level of participants and the activity group allocation. The recruitment in senior centers and trade unions may have led to selecting older adults, who engaged in a physically and socially active life and were, therefore, non-sedentary and above-average in executive function; this may limit the generalization of results. The dichotomization that was chosen to avoid fragmentation and lack of statistical power, though based on cutoffs from international age-related norms, involves the inclusion of medium-active individuals in both groups 

Therefore, future studies are needed to verify whether the present pattern of subjective and autonomic responses to dual-task walking generalizes to older sedentary individuals. 

## 5. Conclusions

In conclusion, the integrated neuroendocrine, behavioral and subjective approach to the response to DT walking situations by aging individuals, who are more or less active, might help detect those at higher risk of developing gait dysfunction or dependence. Also, it might inform the design of appropriate intervention strategies. The promotion of physically active habits could, beyond the general well recognized physical and mental health benefits, specifically contribute to the ability of aging individuals to align the perception of task difficulty to their true ability to perform it efficiently and safely. Moreover, when embedding DT training into physical activity designed for older individuals, it should not only be a matter of progression in the ability to share common resources between locomotor and cognitive task challenges. Rather also of progression in the ability to detect changes in gait stability as a function of the perceived effort and stress response to DT walking challenges. From this perspective, the present line of research may help target the subjectively optimal challenge point in learning of motor-cognitive tasks that mirror the postural control and DT demands of everyday life [18].

## Figures and Tables

**Table 1:** Participants’ background characteristics: gender, anthropometric data, physical activity (daily steps), education, cognitive executive function (Δ Trail Making), number of medications taken and presence of conditions/diseases, retirement, smoking and alcohol habits. Cut-off for lower active (low/somewhat active): ≤9999 steps/day; for higher active (active/highly active): ≥10,000 steps/day.

	Low/Somewhat Active (*n* = 36)	Active/Highly Active (*n* = 40)
**Gender**		
Female (*n*)	16	18
Male (*n*)	23	20
**Height** (m)	1.66 ± 0.11	1.66 ± 0.09
**Body mass** (kg)	77.8 ± 13.4	73.2 ± 12.7
**BMI** (kg·m^−2^)	28 ± 3.5	26.7 ± 4
**Steps** (mean ± SD)	7971 ± 1480	13,766 ± 2479
**Educational level**		
<High school (*n*)	11	6
High school (*n*)	17	24
College (*n*)	11	8
**Δ Trail Making** (s)	54.12 ± 45.42	46.38 ± 31.52
**Medications** (*n*)	4.2 ± 2.7	2.8 ± 2.2
**Diseases** (*n*)	2.2 ± 2.1	1.4 ± 1.9
**Retirement**		
Yes (*n*)	31	27
No (*n*)	8	11
**Smoking**		
No (*n*)	21	18
In the past (*n*)	13	18
Yes (*n*)	5	2
**Alcohol**		
No (*n*)	15	17
Occasionally (*n*)	24	21

**Table 2:** Correlations between dual-task effects (DTE) on performance (gait: CV speed; working memory: WM index), autonomic response (AUC_G_, Pearson’s r) and subjective effort perception (RPE, Spearman’s rho) in all, lower and higher active participants.

		CV Speed	sAA (AUC_G_)	RPE
**DTE of the cognitive task on gait performance(DT–ST_HWS_**)			−0.168 (all)	0.170 (all)
	**CV speed**	-	−0.328 (low-a)	−0.044 (low-a)
			0.336* (high-a)	0.454** (high-a)
			
				−0.141 (all)
	**sAA (AUC_G_)**		-	−0.225 (low-a)
				−0.058 (high-a)
			
	**RPE**			-
		**WM index**	**sAA (AUC_G_)**	**RPE**
**DTE of the gait task on cognitive performance (DT–ST_RNG_)**			0.060 (all)	−0.266* (all)
	**WM index**	−	0.272 (low-a)	−0.079 (low-a)
			−0.232 (high-a)	−0.466** (high-a)
			
				0.197 (all)
	**sAA (AUC_G_)**		-	0.068 (low-a)
				0.319* (high-a)
			
	**RPE**			-

**Note.** DT: dual-task; ST_HWS_: single-task walking at habitual walking speed; ST_RNG_: single-task performance of random number generation; low-a: lower active; high-a: higher active. Significant correlations are highlighted in bold; * *p* < 0.05; ** *p* < 0.001.
